# How do patients and healthcare professionals experience foot examinations in diabetes care? – A randomised controlled study of digital foot examinations versus traditional foot examinations

**DOI:** 10.1186/s12913-024-11674-w

**Published:** 2024-11-12

**Authors:** Ulla Hellstrand Tang, Roy Tranberg, Leif Sundberg, Isabella Scandurra

**Affiliations:** 1 Department of Prosthetics and Orthotics, Sahlgrenska University Hospital, Falkenbergsgatan 3, Gothenburg, 412 85 Sweden; 2 Department of Orthopaedics, Institute of Clinical Sciences, at the Sahlgrenska Academy at the University of Gothenburg, Gothenburg, Sweden; 3 Gothenburg Diabetes Association, Gothenburg, Sweden; 4 Department of Informatics, School of Business, Örebro University, Örebro, Sweden

**Keywords:** Diabetes mellitus, User-centred design, Diabetic neuropathies, Podiatry, Self-care, Diabetic foot, EHealth, Orthotics, Implementation science, Clinical decision support systems

## Abstract

**Background:**

Digital solutions in healthcare can facilitate and improve care. However, the experiences and the usefulness of using either digital foot examinations or traditional foot examinations need to be evaluated.

The aims of the study were to evaluate:

1) The differences in patient experiences, having their foot examined supported by the Clinical Decision Support System as compared with having their foot examined in traditional practice,

2) How healthcare professionals, by using the digital tool, experienced the routine compared with performing the foot examination as in traditional practice.

**Methods:**

Of a total of 141 patients, 100 patients with diabetes were single-blind digitally randomised to one of two parallel arms: having their foot examined by a healthcare professional using a digital tool (*n* = 47) or having their foot examined as in traditional practice (*n* = 53) at the Department of Prosthetics and Orthotics at Sahlgrenska University Hospital, Gothenburg, Sweden. Patients filled in a modified version of the National Patient Survey and the Orthotics and Prosthetics Users’ Survey at study end. Two healthcare professionals, working at a Department of Prosthetics and Orthotics, answered surveys regarding the interaction between the patient and the certified prosthetist and orthotist.

**Results:**

Patients, aged 66 ± 13 years, perceived a high level of satisfaction with the service at the department, regardless of the method used. No significant differences between groups were found when evaluated by 27 questions in the National Patient Survey or by the Orthotics and Prosthetics Users’ Survey, with scores of 67.17 ± 12.18 vs. 66.35 ± 16.52 (p = 0.78) for the intervention and control group respectively. For the same patient that healthcare professionals foot examined, the risk class was fully obtained when the risk to develop foot ulcers was assessed by using the digital tool, whereas only 2% of the patients were classified when foot assessed in traditional practice.

**Conclusions:**

Regardless of the method used for the foot examination, patients perceived a high level of satisfaction with the services at the Department of Prosthetics and Orthotics. All the patients were risk classified in the intervention group. The healthcare professionals found that, by using the Clinical Decision Support System, the foot examination was structured and followed clinical guidelines. Furthermore, the documentation in the electronic health record was thorough, even though further improvements, such as integration with co-existing health record systems, were requested.

**Trial registration:**

Clinical Trials NCT03088566, Registered 23 March 2017.

**Supplementary Information:**

The online version contains supplementary material available at 10.1186/s12913-024-11674-w.

## Background

Digital solutions in healthcare can facilitate, provide structure and improve the recommended foot care [1–3]. Annual structured foot screening and timely prevention, as well as interdisciplinary care, are needed in order to improve the quality of life for patients with diabetes (PDs) at risk of developing diabetic foot ulcers (DFUs), and also in order to reduce high healthcare costs for the treatment of DFUs [4]. In contrast, the lack of a structured foot examination, for PDs, increases the risk of developing DFUs and, taken further, of needing an amputation of the lower extremities.

In Sweden, the national diabetes guidelines are developed by the National Board of Health and Welfare [5]. The provision of healthcare is, however, the responsibility of the 21 semi-autonomous regions, and each region decides whether and when a clinical decision support system (CDSS) should be used [6]. Each of the 21 health regions in Sweden has several municipalities under its jurisdiction and healthcare is organised between region and municipality to meet the needs of the inhabitants [7]. As a result, the care of the feet of elderly PDs in need of social care and healthcare is delivered by the 291 municipalities in Sweden.

All healthcare professionals (HCPs) are obliged to perform a thorough foot examination before they discuss a care plan with the patient [7, 8]. This requirement also applies to certified prosthetists and orthotists (CPOs). Medical doctors are, however, responsible for the prevention and care of DFUs [9–11]. This means that medical doctors are responsible for performing, at the very least, an annual foot examination and risk stratification of the feet prior to a care plan, aimed at promoting good foot health, being presented to the patient as the start of an appropriate intervention [11, 12].

The feet of 27% of PDs nationwide were not examined, according to the Swedish National Diabetes Register [13]. Regarding older PDs the situation was even worse, 82% of the municipalities in Sweden reporting (in 2019) that they did not offer PDs in elderly care an annual foot examination [14]. As a result, neglected, elderly people living in nursing homes in the municipalities run a high risk of developing DFUs due to limited access to an annual foot examination, non-awareness of the current clinical guidelines and a lack of routines specifying how to take care of the PDs’ feet [12].

At the Department of Prosthetics and Orthotics (DPO) at Sahlgrenska University Hospital in Gothenburg, an initiative started in 2010 to create software (D-Foot, Region Västra Götaland [VGR] Gothenburg) to facilitate the completeness of the foot examination and risk stratification [15]. This CDSS was developed and tested in 2014–2015, and its validity, inter-rater and intra-rater reliability were found to be good and acceptable. An iterative process involving the refinement of the D-Foot, based on the experiences of eight users (CPOs) and representatives of the patient organisations, was implemented, enabling a second round of evaluation [15].

A brief overview of other applications used in DFU care shows promising results, but these applications (see Supplementary file 1) have mainly been developed to be used in research and have not been relevant or available for use in clinical practice in Sweden due to legal and regulatory restrictions [16]. One example of a successful nationwide implementation is the risk tool, an electronic health record (EHR) supporting the foot evaluation and risk stratification in Scotland [17–19]. This risk tool was developed in 2008 and has been used for clinical improvements, thereby reducing the prevalence of DFUs [19].

The digitalisation of healthcare procedures is prioritised globally and Sweden is no exception [1]. The vision of “Introducing and transforming healthcare towards digitalised work processes” is a complex task and influences the way HCPs work and how patients assess care [20, 21]. HCPs face well-known everyday problems with poor supporting information technology, e.g., applications and printers that do not work properly, unstable systems and multiple logins into multiple systems, not to mention the fact that documentation often needs to be produced multiple times in different systems. This clearly adds stress for the staff who are already overloaded by their main task, providing care for patients. Nevertheless, IT systems are prerequisites when it comes to improving care, given that the IT systems work effectively and are integrated with the main EHR that is being used. In order to support the daily work situations and the stipulated routines, new methods need to be evaluated, taking account of end-users’ experiences [22]. At the time of this study, a CDSS that was appropriate in Sweden with regard to assessing foot status and providing risk stratification in patients with diabetes was the D-Foot [15]. The D-Foot was a regional medical application. The process of development has followed the 9241-210 ISO standard [23] and regulatory demands [16]. The programming, maintenance and management of this CDSS were administered within the regional IT system. However, based on these standards, further evaluation of the advantages as compared with examining the feet in traditional practice was needed. A CDSS may help to prevent DFUs, but before it is ready for implementation, a test of the CDSS is needed [1–3].

The aims of the study were to:

1. Compare the differences in patient experiences, when having their feet examined with the support of a CDSS, compared with traditional practice.
2. Evaluate how HCPs, i.e., the CPOs, experienced using the CDSS routine as compared to performing the foot examination in traditional practice.

## Methods

### Setting

This randomised, single-blind, controlled study, with a two parallel arm design, was conducted at Sahlgrenska University Hospital at the DPO. Patients were randomised to one, of two, study arms [24], receiving different interventions. After randomisation, each participant stayed in their assigned treatment arm for the duration of the study. The evaluation of the users’ experiences (patients and staff) of the foot examination, was completed when the patients received their assistive devices, two to eight weeks after the first visit. The study was conducted during a period of one month, in the autumn of 2017, according to the procedure used for the nationwide National Patient Survey (NPS) [25]. It was approved by the Regional Ethics Committee Review Board of Gothenburg, Sweden, Id. 414–17, 2017–05-22, and performed in accordance with the Code of Ethics of the World Medical Association (Declaration of Helsinki) for experiments involving humans [26]. Signed consent was obtained from all the participants after they had received verbal and written information.

### Participants – patients

The inclusion criteria for the patients were: being diagnosed with diabetes, age 18 years or older and understanding the Swedish language. The patients had a referral to the DPO with the aim of being provided with assistive devices such as insoles, shoes and/or orthotics, aimed at preventing or treating DFUs. The consecutively included patients (*n* = 141) were randomised to either having their feet examined following the routine in the D-Foot (CDSS group) (*n* = 47) or being examined according to the traditional routine (control group) (*n* = 53). The principal investigator (UT) generated the digital random allocation and assigned participants to interventions when they had signed a written consent to participate in the study. Patients (*n* = 41) that did not participate obtained their assistive devices following the traditional routine and are presented in what follows as dropouts.

### Participants – certified prosthetists and orthotists

The CPOs included in the study were skilled at providing patients with diabetes with assistive devices. Prior to the study start, they completed an introduction course (two times lasting two hours) on how to use the CDSS and they were also trained in and made familiar with the study routine. Five CPOs participated in the study. Two of them had worked as CPOs for more than 20 years, two had worked for one to five years and one had worked for less than one year as a CPO. One of the CPOs had previously used a CDSS when he participated in a study [15].

#### Surveys

All the surveys are presented in Table 1.

**Table 1:** Presentation of the survey used in the study

Name/category	Type of questions	Respondent	Supplementary file, number	References
National Patient Survey	Patient-perceived quality and experience of healthcare	Patients	2	[25, 27, 28]
Orthotic and Prosthetics Users’ Survey	To assess how satisfied the patients are with the devices and the services at the DPO	Patients	3	[29–31]
Survey to the certified prosthetists and orthotists	To measure the interaction between patient and healthcare professional	Certified prosthetists and orthotists	4	

All the patients answered a modified version of the NPS (see Supplementary file 2) [25, 27, 28], and the Orthotics and Prosthetics Users’ Survey (OPUS) (see Supplementary file 3) [29–31]. The answers to each of the questions in the NPS, regarding patient-perceived quality and experience of healthcare, could be given on a scale of 1–4 (1 = Very poorly to 4 = Very well and for some questions 1 = Not at all to 4 = Yes, completely). An alternative of *not applicable* was available (see Supplementary file 2). The modified version had a total of 27 questions covering how patients perceived the service at the DPO (see Supplementary file 2). The second survey, the OPUS, is a survey used to assess how satisfied patients are with the devices and the services at the DPO, recommended by the Swedish Orthotic and Prosthetic industry advisory council for the Swedish evaluation of the services delivered at the DPOs (see Supplementary file 3) [32]. The module entitled the Client Satisfaction with Services (CSS) from the Swedish version, previously validated from the English version, was used (see Supplementary file 3) [33]. The CSS consists of ten items rated on a three-level Likert scale (Strongly agree [1], Agree [2] and Disagree [3], with a fourth option of “not applicable”).

After completing the study, the CPOs filled in a survey measuring the interaction between patient and healthcare professional (see Supplementary file 4 and 5). The questions were related to the information they gave to the patient, to how the CPO experienced the visit and time spent performing the foot examinations, and to how the end-users experienced using the system. Using open-ended questions, comments from the CPOs regarding the CDSS were registered. The advantages of open-ended questions are that the respondents have an opportunity for self-reflection and self-explanations of their experiences of using the CDSS [34].

Furthermore, the survey for the CPOs had seven questions that mirrored questions asked of the patients. For example, the patient question Did *you receive enough information about your care/treatment? was* mirrored for the CPO with the question *Did the patient receive enough information about how to perform self-care of the feet?*

### Clinical Decision Support System – the intervention group

Originating from Region Västra Götaland, the CDSS (D-Foot) was introduced with the aim of improving the foot health of patients with diabetes by using a structured foot examination routine at the DPO following the recommendations in the national guidelines [15]. The users were CPOs and PDs. The PDs filled in answers regarding their diabetes and their foot status. The CPOs made the foot examinations as described in the D-Foot.

**Fig. 1:**
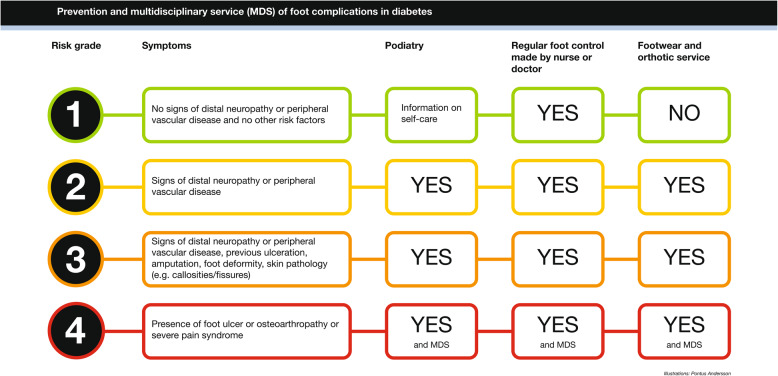
Risk stratification visualised in the D-Foot. Note: MDS = multidisciplinary service. A presentation of the risk classification, the symptoms and the recommended intervention to prevent the development of diabetic foot ulcers as recommended in national guidelines [12]

The intervention group was examined following the routine in the CDSS (vers 1.0, Ortopedteknik, Region Västra Götaland. Each part of the foot assessments has previously been described in detail alongside a description of the patient survey [15, 35]. The D-Foot includes: 1) a patient survey, 2) examinations made by the CPOs, 3) a risk classification, 4) a report on the risk factors present and 5) recommendations about interventions such as podiatry, information about self-care, foot controls and the provision of assistive devices [15]. Based on the patients’ answers in the survey and the assessment made by the CPO, a risk classification (1–4) was generated according to the national risk classification system [12, 36], Fig. 1.

The risk classification and the recommended intervention (podiatry, assistive devices, regular foot controls) were displayed on the screen of a tablet. A printed report was created and handed over to the patient. The result from the D-Foot was then copied and pasted into the EHR at the DPO. At the time of this study, the CDSS was not integrated with the DPOs’ EHR or with the Swedish Diabetes Register.

**Fig. 2:**
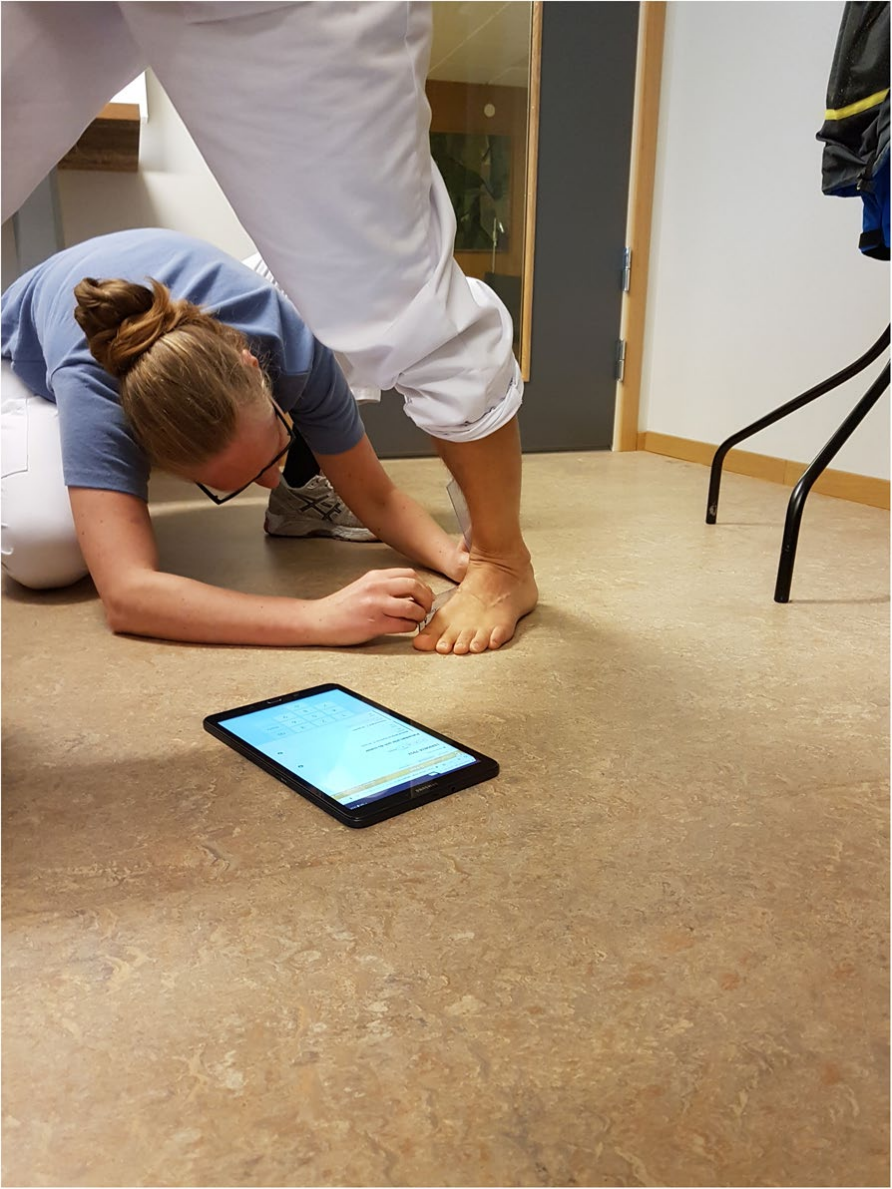
Structured foot assessment with the D-Foot. Note: The structured foot assessment followed the routine in the D-Foot displayed on the screen of the tablet. The CPO measures passive dorsiflexion at the ankle joint

Patients answered the survey at the clinic, using a tablet, Samsung Galaxy Tab A 10.1, and the CPOs then recorded their findings on the same tablet. As previously presented, the CPOs used a goniometer to measure passive maximum dorsal flexion of the hallux at the metatarsal phalangeal joint and passive dorsiflexion at the ankle joint, Fig. 2 [15].

A foot calliper was used to measure foot length, and foot and toe height were measured with a ruler [15]. In addition to the presented features of the D-Foot, the CDSS included a booking section (to register the patient in the system), a database and an administrative part.

### Traditional foot examination - the control group

At the DPO, a foot examination should contain an assessment regarding the presence of peripheral angiopathy/neuropathy, foot deformities, skin pathologies, ulcers, and previous ulcers/amputations, Fig. 1. Based on this assessment, the CPOs should suggest the type of therapeutic footwear that should be prescribed. The documentation takes place after that the patients are provided with therapeutic footwear. The documentation should include the examinations made, the details of the prescription, including advice on self-care, and the prescription made [8, 37]. The execution of the traditional foot assessment depended on the CPO´s individual routine, developed by each of the CPOs.

### Statistics

#### Parallel group trial design

The patients were allocated to one of two, single-blind, parallel arms [24]: having their feet examined using the CDSS or having their feet examined according to general practice. Each patient stayed in their assigned treatment arm for the duration of the study. The digital randomisation was created by the principal investigator (UT) as follows: by using a digital random generator, the two groups were created and handled as pairs. Either the first patient was included in the intervention group and the second in the control group, or vice versa. The generated series could be: CDSS group – control group, control group – CDSS group, CDSS group – control group, etc. The CPOs were blinded to the randomisation.

Descriptive statistics were used to describe the age and gender of the participating patients. Dichotomous variables are presented as numbers and continuous values are reported as the mean, standard deviation (SD). Descriptive data, including foot status and the risk classification for participants in the intervention group, were analysed based on the data stored in the D-Foot software at VGR. For the control group, the risk classification was based on the recording in the EHR.

In the NPS, a separate chi-square test was conducted for each question. The differences between the intervention group and the control group, based on the OPUS, were analysed with an independent samples t-test and a 95% confidence interval.

Responses to the OPUS are analysed separately for each module, yielding an “OPUS score”, a summary measure on a scale of 0–100 [29–31]. The OPUS has been developed and validated using Rasch analysis, a statistical method whereby the summed score is converted to Rasch values. Conversion can be achieved using conversion tables or key form tables. Note that responses to many questions must be reversed before entering them in the tables, so that a high score corresponds to high function, satisfaction, or quality of life, and a low score corresponds to low function, satisfaction, or quality of life. At the bottom of each table, the Smallest Detectable Difference (SDD) is indicated. This is the smallest difference between two OPUS scores that needs to be achieved for us to believe that the score difference reflects a real change and not just random variations.

Seven questions in the NPS were mirrored in the survey that was answered by the CPOs, and one of the questions was visualised by a bar chart.

A chi-square test was used to calculate the differences in gender between the group of patients not included (dropouts) and the group of patients included. An independent samples t-test was used to calculate the differences in age between the participants that were included and the patients that did not participate.

#### Power analysis

The study had a two-group parallel design. The assumptions relating to the number of patients to include, 58 in each group, were based on a power of 0.8 and a difference between groups regarding the proportion of positive answers to the NPS (the intervention group: 0.75 vs. the control group: 0.50).

## Results

**Fig. 3:**
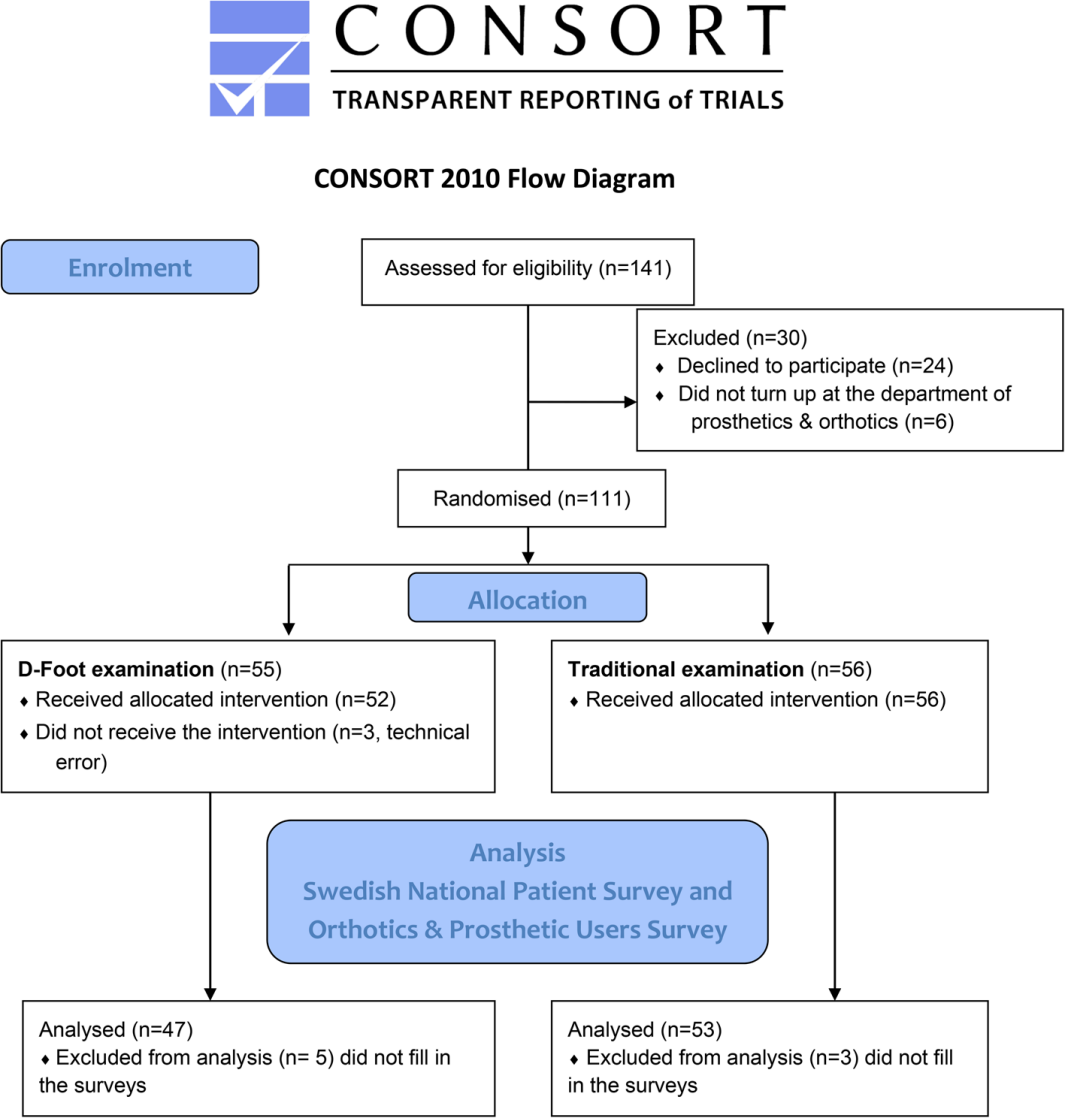
Flow diagram of the patients included and the dropouts

A total of 141 patients were asked to participate and 100 patients agreed to participate in the study, Fig. 3.

The reasons for not participating were that participants did not turn up for the appointment at the DPO or did not want to participate. The intervention group included 47 patients and the control group 53 patients.

The mean age of the group (*n* = 100) was 66 ± 13 years. Patients not included (*n* = 41) had a mean age of 65 ± 14 years, 21 were women and 20 men, and the dropouts did not differ in age or gender compared with the group of patients included. The intervention group had an age of 66 ± 14 years, 26 were women and 21 men, Table 2. The control group had an age of 68 ± 12, 20 were women and 33 men, Table 2.

**Table 2:** General descriptions of the intervention group examined with the Clinical Decision Support System, the D-Foot

	**Missing (n)**	**Numbers**	**Mean (SD)**
**A. General**			
Type 1 diabetes		11/46	
Type 2 diabetes		35/46	
Diabetes, other types		n/a	
Duration (yrs)			18 (15)
Age (yrs)			66 (14)
First visit/revisit to the DPO		70 vs. 30	
HbA1c (mmol/mol), self-reported			62 (20)
Height (cm)			169 (10)
Weight (kg)			85 (25)
BMI (kg/m2)			30 (7)
Medication for high blood pressure		31/46	
Medication for heart disease	1	17/46	
Smoker	2	8/46	
Use snuff		4/46	
Do you think that you have normal sensation in your feet?	1	26/46	
Do you feel that you can walk normally?		24/46	
Relative time sitting/lying vs. standing/walking (0 = sitting/lying during time awake and 100 = standing/walking during time awake)			52 (29)
**B. Preventive interventions, the last 12 months**			
Have you been referred to podiatry?		28/46	
Have your feet been examined by a doctor or a nurse?	1	35/46	
Did you get enough information about self-care?		30/46	
Have you previously received footwear or assistive devices?		22/46	
**C. Occupation**			
Working		6/46	
Student		0/46	
Retired		28/46	
Other		7/46	

Categorical variables are presented as numbers and percentages (%) and continuous variables are presented as the mean and standard deviation (SD). Missing values are due to technical errors in the CDSS. Therefore, a total of 46 answers are found in Table 2

Out of 47 patients in the intervention group, 46 were registered in the CDSS. The general descriptives, origin from the CDSS, showed that 28 were retired and 11 had type 1 diabetes, Table 2. Thirty patients in the intervention group had previously visited the DPO. Patients in the intervention group had a duration of diabetes of 18 ± 15years, BMI 30 ± 7, and 31 were on medication for high blood pressure. Similar descriptors were lacking in the EHR for the control group.

In Table 2, preventive interventions, such as access to podiatry, information about self-care and foot examinations, are presented for the intervention group. Information regarding preventive interventions for the control group was not registered manually in the EHR.

Due to a technical error in the CDSS that was used, one patient could not be examined using the digital routine. In Table 3, the number of patients in each of the risk classes is presented: 46 PDs of a total of 46 PDs in the intervention group and two PDs from 53 PDs in the control group.

**Table 3:** Categorical measurements of risk factors for developing diabetic foot ulcers

**Risk level**	**Intervention Group** **(n/total registered)**	**Control Group** **(n/total registered)**
	Both feet	Both feet
Risk level 1	0/47	
Risk level 2	3/47	
Risk level 3	38/47	2/2
Risk level 4	5/47	

Categorical variables are presented as numbers from total. In the control group, 51 patients with diabetes were not risk classified. Missing value is due to technical errors in the CDSS. Therefore, a total of 46 patients were risk classified int the intervention group

### Patients’ experiences – the NPS

In Table 4, the number of patients’ answers is presented for each of the 27 questions in the NPS.

**Table 4:** Results from the modified version of the National Patient Survey (NPS) regarding how satisfied the participants were with the service at the Department of Prosthetics and Orthotics

**Question NPS, score 1–4 and not applicable**	**N**	**Intervention** **n/total**	**Control**	***p*-value**
NPS 1	100			0.75
3		2/47	3/53	
4		45/47	50/53	
n/a		0	0	
NPS 2	97			0.17
2		0/46	2/51	
3		1/46	4/51	
4		45/46	45/51	
n/a		1	2	
NPS 3	98			0.16
2		0/46	1/52	
3		0/46	3/52	
4		46/46	48/52	
n/a		1	1	
NPS 4	95			0.11
2		0/45	2/50	
3		1/45	5/50	
4		44/45	43/50	
n/a		2	3	
NPS 5	91			0.57
2		0/44	1/47	
3		2/44	3/47	
4		42/44	43/47	
n/a		3	6	
NPS 6	96			0.05
3		0/46	4/51	
4		45/45	47/51	
n/a		2	2	
NPS 7				0.08
3		1/45	6/52	
4	97	44/45	46/52	
n/a		2	1	
NPS 8	97			0.01
3		1/45	10/52	
4		44/45	42/52	
n/a		2	1	
NPS 9	82			0.06
1		7/37	4/45	
2		0/37	1/45	
3		2/37	11/45	
4		28/37	29/45	
n/a		10	8	
NPS 10	83			0.30
1		0/38	2/45	
2		0/38	1/45	
3		8/38	13/45	
4		30/38	29/45	
n/a		9	8	
NPS 11	87			0.36
2		1/39	2/48	
3		4/39	10/48	
4		34/39	36/48	
n/a		8	5	
NPS 12	82			0.34
1		3/39	2/43	
2		0/39	1/43	
3		5/39	11/43	
4		31/39	29/43	
n/a		8	10	
NPS 13	68			0.71
1		4/31	6/37	
2		1/31	3/37	
3		3/31	5/37	
4		23/31	23/37	
n/a		16	16	
NPS 14	77			0.92
1		4/35	3/42	
2		3/35	3/42	
3		6/35	8/42	
4		22/35	28/42	
n/a		12	11	
NPS 15	95			
3		2/45	4/50	
4		43/45	46/50	0.48
n/a		2	2	
NPS 16	95			
3		0/44	5/51	
4		44/44	46/51	0.03
n/a		3	2	
NPS 17	56			0.21
1		1/29	0/27	
3		0/29	2/27	
4		28/29	25/27	
n/a		18	26	
NPS 18	38			0.66
1		0.1; 1/17	0.0; 1/21	
3		0.0; 0/17	0.0; 1/21	
4		16/17	19/21	
n/a		30	32	
NPS 19				
4	88	43/43	45/45	N/A
n/a		4	8	
NPS 20	90			
3		0/46	5/44	
4		46/46	39/44	0.02
n/a		1	9	
NPS 21	24			0.22
1		1/12	0/12	
3		0/12	2/12	
4		11/12	10/12	
n/a		35	41	
NPS 22	93			0.55
2		0/45	1/48	
3		3/45	2/48	
4		42/45	45/48	
n/a		2	5	
NPS 23	93			0.28
2		0/45	1/48	
3		3/45	7/48	
4		42/45	40/48	
n/a		2	5	
NPS 24				
3		0/47	3/51	
4	98	47/47	48/51	0.09
n/a		0	2	
NPS 25	50	27	23	0.23
1		13/20	14/30	
3		0/20	3/30	
4		7/20	13/30	
n/a		27	23	
NPS 26	96			0.81
2		1/45	1/51	
3		5/45	8/51	
4		39/45	42/51	
n/a		2	2	
NPS 27	100			0.56
2		0/47	1/53	
3		1/47	2/53	
4		46/47	50/53	
n/a		0	0	

Intervention: the group of patients with diabetes whose feet were examined and registered in the Clinical Decision Support System

Control: the group of patients with diabetes whose feet were examined as in traditional practice

N is the number of answers. Scoring 1–4, i.e., the patients had the opportunity to score the answers from 1–4 where 1 = very poorly and 4 = very well and n/a = not applicable. For some questions 1 = Not at all to 4 = Yes, completely and n/a = not applicable was available

The analysis did not take account of the fact that multiple comparisons exist

Missing values are due to patients failing to fill in all the answers. Of the 27 questions in the modified version of the NPS, no differences between groups were found, Table 4. The intervention group answered four questions (6, 8, 16, 20) positively, compared with the control group. These questions were:

- 6. Did you feel that you were treated with respect and dignity regardless of: sex, gender identity or expression, ethnic affiliation, religion or other beliefs, disability, sexual orientation or age? (*P* = 0.05)
- 8. Were you involved in the decisions regarding your care/treatment to the extent you wished? (*P* = 0.01)
- 16. Were you given an opportunity to ask the questions you wanted? (*P* = 0.03)
- 20. If any examinations were made, did the certified prosthetist and orthotist explain the results in a way that you understood? (*P* = 0.02).

### Patients’ experiences – the OPUS

**Fig. 4:**
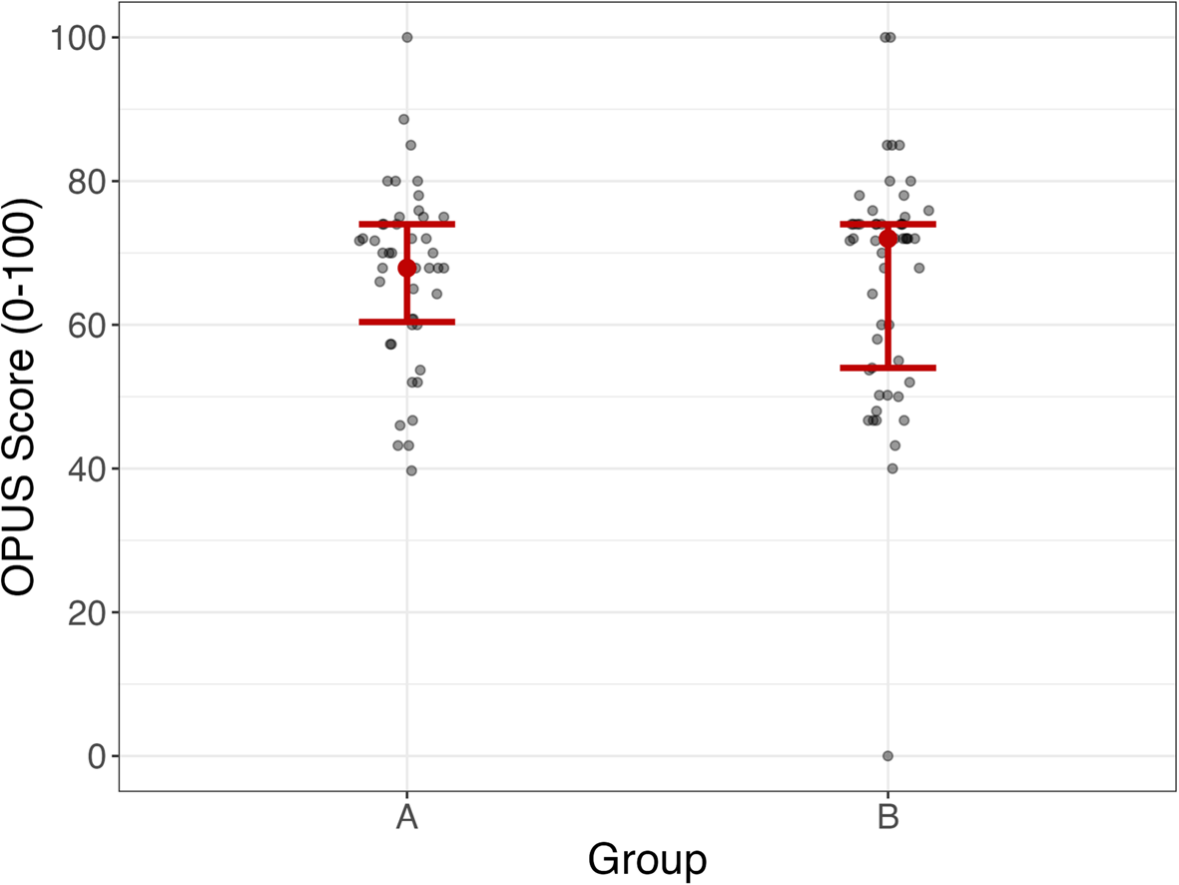
Differences in the way patients experienced the visit to the Department of Prosthetics & Orthotics, measured with the Orthotic and Prosthetic Users’ Survey. Note: The x -axis represents the two groups. Group A = the intervention group and group B = the control group. The y -axis shows the OPUS scores reported by the participants. Presentation of the distribution of answers for the intervention grop (A, *n* = 47) and the control group (B, *n* = 53). The red lines indicate the 25th and the 75th percentiles. No significant difference between groups, regarding the OPUS score, 0.83 (p = 0.78). One individual in the control group had a score of”0″ because the participant answered “1″ for all 10 questions

No statistical differences between groups were found regarding patients’ experiences and satisfaction measured with the OPUS, 67.17 ± 12.18 vs. 66.35 ± 1 6.52 for the intervention group and control group respectively. The difference between groups was 0.8 with a 95% CI of -4.99 to 6.65, effect sizes Cohen’s d = 0.05, Fig. 4. Missing values were due to patients failing to fill in all the answers.

### CPOs’ experiences

The experiences of the different types of foot examination, with the CDSS or according to traditional practice, are presented in Table 5.

**Table 5:** Experiences of the interaction between the patient and the certified prosthetist and orthotist (CPO). The survey is an overall description of how the CPOs experienced the interaction, regardless of the method used during foot examination

**Questions**	**1** ***Not at all***	**2**	**3**	**4** ***Yes, completely***
	N	N	N	N
11. Did the patient receive enough information about how to take care of their feet?	1			4
12. Did the patient receive enough information about their care/treatment?				5
13. Did you give enough information about where the patient should go if they needed help or had additional questions after the visit?			2	3
14. Did the patient receive enough information about possible risks of using the assistive device?	1		2	2
15. Did the patient receive enough information about warning signs to be aware of regarding their illness/health condition or your assistive device?			2	3
16. Was there enough privacy when you and the patient talked about their condition or treatment?				5
17. Did you explain the results of the D-Foot in a way the patient understood?		1		4
18. Did you give the patient oral information about the assistive devices?			2	3
19. Did you give the patient written information about the assistive devices?		2	1	2

After completing the study, the CPOs answered a questionnaire on the information they had given to the patients and how the CPOs experienced their interaction with the patients. The CPOs answered the questions on a scale from 1 (Not at all) to 4 (Yes, completely) or not applicable. The option of not applicable was not selected by any of the CPOs and is therefore excluded from the table

The CPOs scored the interaction with their patients as “3–4”, interpreted as good, Table 5. Four of five CPOs estimated that the time spent on the patient’s visit was 46–90 min and one reported 31–45 min, Table 6.

**Table 6:** Experiences from the digital foot examination

**Questions**
20. Hur lång tid tog patientbesöket? Räkna inte in tiden för att journalföra besöket i Pilot. Time the patient visit took. Exclude the time taken to document in the electronic medical record (EMR)
1) < 30 min
2) 31–45 min. = 1 CPO
3) 46–60 min. = 4 CPOs
4) > 60 min
21a. Hur lång tid tog det att journalföra i Pilot när du undersökte fötterna enligt D-Foot metoden? Time spent documenting in the EMR after finishing the digital foot examination?
1) < 5 min. = 1CPO
2) 6–10 min. = 4 CPOs
21b. Hur lång tid tog det att journalföra i Pilot när du undersökte fötterna enligt gängse praxis? Perceived time spent documenting in the EMR after finishing the traditional foot examination?
1) 6–10 min. = 4 CPOs
2) = 11–15 min. = 1 CPO
Hur lång tid tog det att göra eventuella sko- och materialbeställningar? Time spent ordering material and shoes?
1) < 5 min. = 2 CPOs
2) 6–10 min. = 3 CPOs

The time estimate did not include the time needed to document patient status in the EHR. The registration in the EHR at the DPO was made more rapidly when the results from the digital foot examination using the D-Foot were copied and pasted in the EHR, < 5 min (*n* = 1 CPO) and 6–10 min (*n* = 4 CPOs), compared with the traditional examination, 6–10 min (*n* = 4 CPOs) and 11–15 min (*n* = 1 CPO).

Of a total of five CPOs, one of the CPOs made suggestions, in free text, on how to improve the D-Foot application, Table 7.

**Table 7:** Comments from a certified prosthetist and orthotist (CPO) regarding the Clinical Decision Support System, the D-Foot

**Comments in Swedish**	**Comments in English**
Vid vissa hälsotillstånd svårt att gå vidare i programmet	It is difficult to continue the programme in some states of health
Svårt att svara "rätt" på alla frågor då alla patienter ej fått sitt hjälpmedel	Difficult to answer all the questions in a relevant way because some patients had not received their equipment [not the D-Foot but the other questions to CPO]
Lägg till en fritextruta för annan väsentlig information såsom andra sjukdomar, sårplacering, amputationsnivå mm	Add a field in the D-Foot, for additional information, e.g., other diseases, location of the ulcer, level of amputation, etc
Sidan 4 hade för mig fungerat bättre enl: 1. fotlängd 2. fotbredd 3. maximal tåhöjd 4. fotledsrörlighet	Page 4 (in the D-Foot) would have worked better for me as follows: 1. Foot length 2. Foot width 3. Maximum toe height 4. Passive range of movement at the ankle joint
Det känns tryggt att veta att allt som enligt forskning kan vara riskfaktorer har undersökts samt att inget missas till journalföringen	It feels good to know that risk factors, identified in research, have been examined and that nothing will be missed in the medical recording
Att endast fynd förs in i journalen. Det blir mer relevant för OI	Better if only the findings [risk factors, the authors’ interpretation] are registered in the medical record system. This is more relevant for the CPO
Lägga till mer fotdeformiteter. Mer test för korrekt åtgärd, trycktest över framfot, Coleman blocktest	Add more examinations regarding the presence of foot deformities. Additions before suggesting an adequate intervention, e.g., the Coleman block test

In Table 7, the improvements, such as adding fields to make registrations of other diseases and foot deformities and changing the order of the examinations, are presented.

### Patients’ vs. CPOs’ experiences of the service at the DPO

**Fig. 5:**
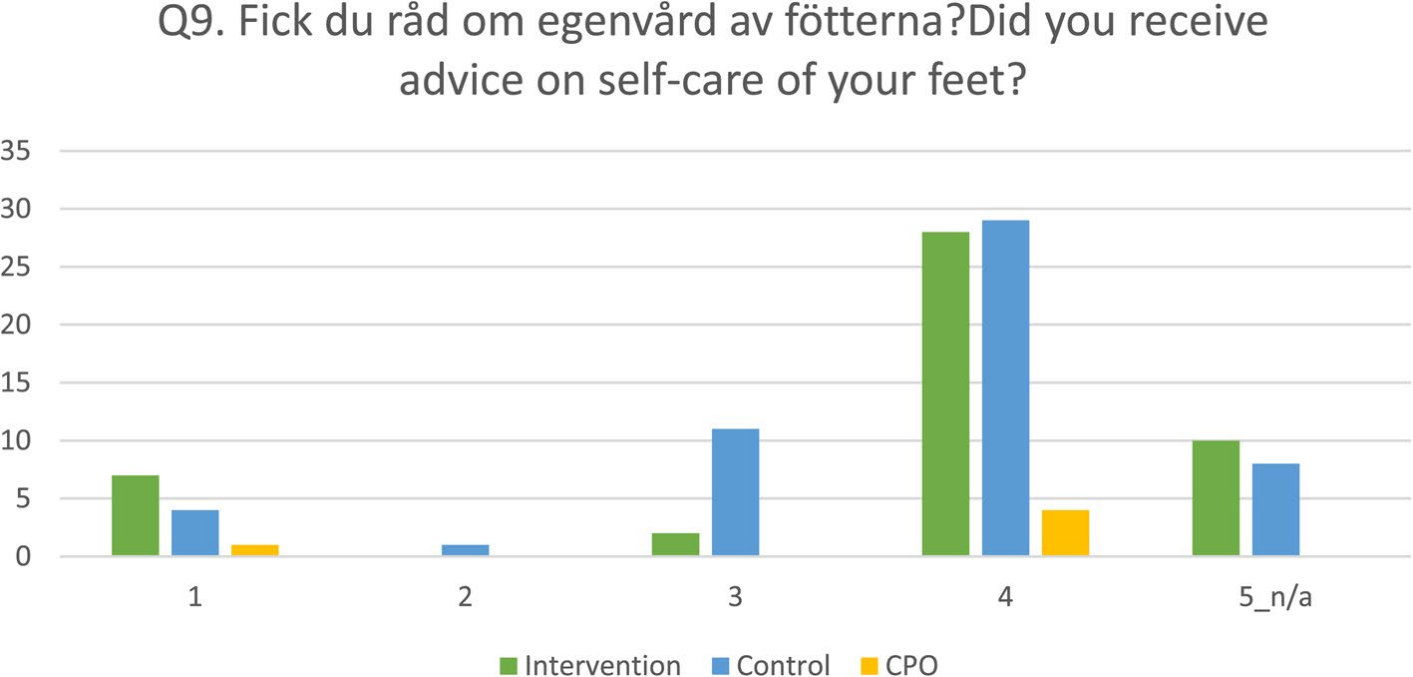
An example of how patients and CPOs perceived the service at the Department of Prosthetics & Orthotics. Note: The figure shows the answers that patients in the intervention group (CDSS), *n* = 47, and the control group (Control), *n* = 53, and the certified Prosthetists and Orthotists (CPOs), *n* = 5, gave to the question “Did you receive (give) advice on self-care of the feet?”. The answers have a scale from 1 (Not at all) to 4 (Yes, completely) or not applicable (n/a)

Seven questions were answered by all the patients in both groups and the CPOs, respectively, Tables 4 and 5. For the seven questions, in all three groups, the most frequent response was “4 = very well/yes, completely”. In Fig. 5, one of the questions is visualised by a bar chart.

Question 20 (“*Om det gjordes några undersökningar, förklarade ortopedingenjören resultaten på ett sätt som du förstod? If any examinations were made, did the certified prosthetist and orthotist explain the results in a way that you understood?* “) received more “not applicable” answers in the control group compared with the intervention group.

## Discussion

The most important finding when studying the development of a structured digital routine for foot assessment (CDSS) for patients with diabetes, is the high level of satisfaction with the services at the DPO. Similar results were found, measured by a modified version of the National Patient Survey and by the Orthotic and Prosthetic Users’ Survey, for the intervention group and the control group. The high level of satisfaction, regardless of the method, can be explained by biases such as the Hawthorne effect, meaning that study participants might change their behaviour or perceptions to be more positive as a consequence of the attention they receive, irrespective of the intervention [39, 40]; the involved CPOs being highly experienced; the DPO being friendly and clean; and, finally, the level of care given by the DPO being already at a high level. No adverse events occurred. In clinical practice where a high level of care already exists, potential improvements are difficult to measure in an RCT. Moreover, the method used could have had limitations when it came to measuring satisfaction with the service at the DPO. Before discussing the result, regarding how satisfied the patients were with the services at the DPO, the advantage of a digital routine is discussed.

In this study, like previous studies [17, 35], several advantages are obtained when using a CDSS, the D-Foot. They are: the foot assessment and the risk stratification follow national clinical guidelines [12, 41]; the risk stratification is generated by the system and is therefore unbiased [15]; the registration in the EHR at the DPO was made faster when the results of the digital foot examination were used as a basis for the documentation, < 5 min (*n* = 1 CPO) and 6–10 min (4 = CPOs), compared with a traditional examination, 6–10 min (*n* = 4 CPOs) and 11–15 min (*n* = 1 CPO). In general, our findings are in line with previous literature reporting that using a structured routine in foot screening leads to an improvement of care aimed to prevent DFUs [17, 18, 38, 42]. Implementation, however, is surrounded by regulations and frameworks for respectively county and/or country [43]. The added value when moving towards a person-centred care is our reporting from the users’ perspective. The satisfaction of using the CDSS was high in the current study.

Moreover, information regarding the use of medicine for high blood pressure and/or heart diseases and regarding perceived preventive interventions (podiatry and information on self-care of the feet), is useful prior to a discussion of the care plan. The descriptors registered in the D-Foot, such as profession, use of medicine for high blood pressure/heart disease and interventions to prevent DFUs. are of great importance. In the present study, we found that this information was not registered for the control group.

It is noteworthy that patients in the intervention group reported limited access to podiatry or information about self-care of the feet, despite national clinical recommendations for diabetes [12]. Moreover, one-third of the patients said that their feet had not been examined during the last 12 months.

The results in Table 3 reveal that, by using the CDSS, 100% of the patients were risk classified, in contrast to the control group where only 2% were risk classified. Moreover, 83% of the patients in the intervention group were classified as risk grade 3. The patients in risk grade 3 had one or more of the following risk factors: foot deformities, skin pathologies, previous DFU/amputation and signs of angiopathy/neuropathy, and were in great need of prescribed footwear. If foot deformities, such as hallux valgus, limited range of motion in foot joints and gait deviation, are not assessed, or skin pathologies are neglected, there is a risk that the prescribed footwear will be based on weak reasons and that advice on self-care will be left out [8, 37]. Eleven per cent had DFUs and were classified as risk grade 4. The lack of risk classification in the EHR for the patients in the control group is serious. The negative consequences for the patient are severe if the risk classification is not registered. If an ulcer (risk grade 4) is not noticed and therefore not registered, it is a great concern that the adequate offloading device will not be prescribed, thereby prolonging the healing period [44].

The nationwide use of a CDSS, like the use of the risk tool in Scotland, is necessary to ensure patient security, and we therefore recommend the full implementation of the CDSS at the DPOs in Sweden. This would increase the proportion of patients whose feet are examined, who are risk classified and who are prescribed the appropriate therapeutic footwear or orthoses. Furthermore, the meta-data from the CDSS create an opportunity for audits and the continuous improvement of foot care [19, 38].

In the current study, no adverse events occurred as a result of using the CDSS. It was not cumbersome (no pain, no complications) for the patient, but nor was the traditional method that was evaluated in this study.

### Refinements of the CDSS

Further developments in the co-design [45, 46] of the CDSS, following the ISO standard, were requested [16]: add fields to make registrations of co-morbidities, specify the level of amputation, specify the location of the ulcer, change the order of measurements and add measurements of foot deformities, e.g., the Coleman block test [47].

To be able to promote the patient’s integrity, self-determination and participation in the prescription of assistive devices for patients at risk of developing DFUs, the integration of the EHR in primary care, municipality care and specialist care is needed. At the time of this study, the DPO used its own EHR, which was not integrated with the EHR in primary care or other units at Sahlgrenska. This means that, if a person moves to another location, the EHR, with information on the patient’s risk stratification and risk factors, does not follow the patient. Integration between the DPO’s EHR, the EHR used in the municipality and the EHR used in primary care and specialist care, is therefore needed. One registration is required, meaning that if, for example, blood glucose levels and the use of medicine for high blood pressure are registered in the EHR, as the patients are examined in primary care, this registration should also be available in the EHR at the DPO. Bridging healthcare borders between primary and specialist care, in the care of DFUs, is more complex, as described by Koltveit et al., but it is possible [48, 49]. Like Koltveit et al., the authors of the present study plan to involve stakeholders (policy makers, healthcare managers, IT departments, patient representatives and clinicians) in the implementation process, as they are the cornerstones of success [48]. The goal is to create integrated care across different levels of healthcare providers [49].

Our results show that important information is missing in the EHR for the control group. By using a CDSS, the CPOs will enhance their skills and documentation will be improved [50]. Moreover, like the findings reported by Koltveit et al., increased skills and knowledge among the HCPs are expected.

A prerequisite for national improvements in the care of DFUs is the integration of the CDSS with quality registers such as RiksSår, the Swedish national quality register for ulcer treatment [51, 52], and the Swedish National Diabetes Register [36]. At the time of this study, these registers did not register foot parameters, nor the number and proportion of patients in each of the risk categories or the underlying risk factors.

In further research, the usability of the D-Foot should be evaluated by all users: patients, CPOs, healthcare managers, IT experts and other healthcare givers [21]. Moreover, a long-term effect of using a CDSS, with a reduction in the number of DFUs in primary care, is expected and should be of interest in future studies. In order to check that preventive interventions reach PDs, the number and proportion of patients who are referred to podiatry should be registered and followed up [12].

### Limitations and strengths

In the current RCT, a number of dropouts were due to patients not turning up at the department, technical errors or the fact that the surveys were not completed. Instead of groups consisting of 58 participants, the final number was 47 for the intervention group and 53 for the control group. A recommendation for future studies is to increase the number of participants by 10–15% compared with the number obtained from the power analysis, to compensate for dropouts. In the current study, the study period was one month, as recommended by the Swedish Association of Local Authorities and Regions when collecting answers with the national patient survey. A prolonged study period would have increased the number of participants.

Some considerations in the study are related to the study participants. Selection bias, meaning that participants with more motivation are anticipated, might lead to a positive score in the answers in the survey.

To strengthen future studies, we recommend that the registrations regarding the time it took to make the foot examination and to make the registration in the EHR, are observed and registered by an independent observer following the CPO during the study. In the current study, each CPO made an approximation of the time taken for these tasks, for the intervention group and the control group respectively, at study end. The registration should preferably be made after each individual appointment. Another improvement is that the DPO should answer the question regarding the interaction with the patient after each individual patient that the CPO has examined, not at study end as a summary registration.

In the NPS and the OPUS, some questions were related to the environment and the waiting time at the DPO. These questions do not appear to be relevant when measuring differences between groups regarding their experiences of the services at the DPO and how PDs experienced the foot examinations. The irrelevant questions were: question 26 in the NPS: “*Did you feel that it was clean at the clinic*?” and question 3 in the OPUS: “I waited a reasonable amount of time to be seen” [33]. In fact, the patients that participated in the current study did not wait a long time in the waiting room because the principal investigator rapidly took care of the patient. Moreover, the questions 13, 14, 17, 18 and 21 were not relevant in a DPO setting, despite being relevant to healthcare in general. Many participants answered these questions with “not applicable!”. Even if the NPS, the nationwide survey used to measure patient-perceived quality and experience of healthcare, was recommended [25], we suggest that the OPUS with the Client Satisfaction with Services module (CSS) should be used in further studies, as a tool to assess how satisfied patients are with the devices and the services at the DPO [32, 33], in addition to qualitative methods for studying usability [21].

To strengthen similar study results, we recommend that surveys are filled in at the clinic on the final visit. In this study, some patients left the DPO without filling in the NPS or the OPUS. To collect their answers on the surveys, an envelope including a postage-paid reply envelope was sent to them with an information letter asking them to fill in the survey. However, three patients did not return their answers.

Regarding statistics, the results of the 27 questions in the NPS in Table 4 do not take account of the fact that multiple comparisons are made. As a result, the original alpha values are reported. Detecting whether a new intervention is superior to the traditional way of working in existing high-level care, is difficult with traditional RCTs [53]. In future studies, we recommend that equivalence studies be considered, as an alternative approach to determine whether the new intervention is having similar effects to traditional practice [54]. This suggestion is supported by the findings from the OPUS, showing that the difference between groups was 0.8 with a 95% CI of -4.99 to 6.65, effect sizes Cohen’s d = 0.05, typically interpreted as a small or very small effect. So, instead of proving that the intervention is superior to traditional foot examinations, further studies can focus on the added value of structured foot examinations. Optimally, added values, such as early detection of risk factors, timely prevention, interdisciplinary care, improved quality of life for patients with diabetes and reduced health care costs, should be measured.

## Conclusions

By using a CDSS, patients perceived a high level of satisfaction with the services at the DPO and all the patients were risk classified.

The CPOs found that, by using the CDSS, the structured foot examination was facilitated and followed clinical guidelines.

The documentation from the CDSS to the EHR included necessary information, but further improvements, e.g., integration with the EHR, were requested.

## Supplementary Information

Supplementary Material 1. Overview of apps.

Supplementary Material 2. National patient survey, translated.

Supplementary Material 3. Orthotics & prosthetics users survey.

Supplementary Material 4. Survey to healthcare professionals I.

Supplementary Material 5. Survey to healthcare professionals.

## Data Availability

The dataset analysed during the current study is not publicly available. For detailed information on routes of calculation, please contact the corresponding author.

## Abbreviations

CDSS: Clinical decision support system
CPO: Certified prosthetist and orthotist
DFU: Diabetic foot ulcer
DPO: Department of Prosthetics & Orthotics
EHR: Electronic health record
EMR: Electrical medical record
HCP: Healthcare professional
NPS: National Patient Survey
OPUS: Orthotics and Prosthetics Users’ Survey
VGR: Region Västra Götaland
