# Supplementary material for: How do patients and healthcare professionals experience foot examinations in diabetes care? – A randomised controlled study of digital foot examinations versus traditional foot examinations
Source: BMC Health Serv Res. 2024 Nov 12;24:1387. doi: 10.1186/s12913-024-11674-w (PMC11558827; doi:10.1186/s12913-024-11674-w)
Supplement: Supplementary file 1 — Supplementary Material 1. Overview of apps. [file 12913_2024_11674_MOESM1_ESM.docx]

**Supplementary file 1**. An overview of applications used to facilitate the prevention and care of diabetic foot ulcers

| Details | Intended use | Features | Primary users | Secondary users | Integration with EHR | Type | Country | Reference |
| --- | --- | --- | --- | --- | --- | --- | --- | --- |
| M-DAKBAS | To facilitate self-care | Register glucose values and foot observations | Patients with diabetes | s | Not clarified | Research project | Turkey | [46] |
| The risk tool | To predict the risk of foot ulceration | A risk stratification is assessed based on a foot examination | Healthcare professionals | n/a | Yes, a part of the EHR | Implemented in care | United Kingdom/Scotland | [17-19] |
| MyFootCare | To engage patients with diabetes in the healing process of the foot ulcers by setting goals, reminders on self-care and monitoring the healing progress | Take care of the foot ulcer and facilitate engagement by setting goals, reminders on self-care and monitoring the healing progress | Patients | Healthcare professionals | Not clarified | Research project | Australia | [50] |
| Telemedicine | DFU assessment | To assess DFU by telemedicine web platform where photos are taken with a mobile phone + ulcer record from primary healthcare to specialist healthcare for assessment of the ulcer | Primary care nurses | Specialists in healthcare | Not clarified | Research project | Norway | [48, 49] |
| Diabetic foot examination (CDFE) | To improve the completeness and frequency of diabetic foot exams | A standardized documentation form + an electronic reminder | Healthcare professionals | Quality improvers | Not clarified | Research project | United States of America | [38] |
