# Supplementary material for: How do patients and healthcare professionals experience foot examinations in diabetes care? – A randomised controlled study of digital foot examinations versus traditional foot examinations
Source: BMC Health Serv Res. 2024 Nov 12;24:1387. doi: 10.1186/s12913-024-11674-w (PMC11558827; doi:10.1186/s12913-024-11674-w)
Supplement: Supplementary file 2 — Supplementary Material 2. National patient survey, translated. [file 12913_2024_11674_MOESM2_ESM.pdf]

Supplementary file 2. Survey for patients who participate in a study about foot examinations at the Department of Prosthetics and Orthotics

This survey contains questions about your visit to the Department of Prosthetics and Orthotics. Answer the questions by selecting the answer that fits best. If you are unsure, please select the box that feels most accurate. Tick the box like this ☒

How was today's meeting at the Department of Prosthetics and Orthotics?

Answer the questions by selecting the answer that fits best on a scale from 1 (Very poorly or Not at all) to 4 (Very well or Yes, completely).
If you are unsure, please select the box that feels most accurate. Tick the box like this ☒

1) **How were you treated by the healthcare staff who received you upon arrival/at the reception?**

1. Very poorly	2.	3.	4. Very well	5. Not applicable
☐	☐	☐	☐	☐

2) **Did the certified prosthetist & orthotist take into account your own experiences of your illness/your health condition?**

1. Not at all	2.	3.	4. Yes, completely	5. Not applicable
☐	☐	☐	☐	☐

3) **Did the healthcare staff approach you with compassion and care?**

1. Not at all	2.	3.	4. Yes, completely	5. Not applicable
☐	☐	☐	☐	☐

4) **If you asked questions to the healthcare staff, did they respond with compassion and interest?**

1. Not at all	2.	3.	4. Yes, completely	5. Not applicable
☐	☐	☐	☐	☐

5) **If you asked questions to the healthcare staff, did you receive answers that you understood?**

1. Not at all	2.	3.	4. Yes, completely	5. Not applicable
☐	☐	☐	☐	☐

6) **Did you feel treated with respect and dignity regardless of: sex, gender identity or expression, ethnic affiliation, religion or other beliefs, disability, sexual orientation or age?**

1. Not at all	2.	3.	4. Yes, completely	5. Not applicable
☐	☐	☐	☐	☐

7) **Did the certified prosthetist & orthotist include you in the decisions regarding your care/treatment?**

1. Not at all	2.	3.	4. Yes, completely	5. Not applicable
☐	☐	☐	☐	☐

8) **Were you involved in the decisions regarding your care/treatment to the extent you wished?**

1. Not at all	2.	3.	4. Yes, completely	5. Not applicable
☐	☐	☐	☐	☐

9) **Did you receive advice on self-care of your feet?**

1. Not at all	2.	3.	4. Yes, completely	5. Not applicable
☐	☐	☐	☐	☐

10) **Did you receive enough information about your illness/your health condition?**

1. Not at all	2.	3.	4. Yes, completely	5. Not relevant
☐	☐	☐	☐	☐

11) **Did you receive enough information about your care/treatment?**

1. Not at all	2.	3.	4. Yes, completely	5. Not relevant
☐	☐	☐	☐	☐

12) **Did you receive enough information about where to go if you needed help or had additional questions after the visit?**

1. Not at all	2.	3.	4. Yes, completely	5. Not applicable
☐	☐	☐	☐	☐

13) **Did you receive enough information about possible risks with using the assistive device?**

1. Not at all	2.	3.	4. Yes, completely	5. Not applicable
☐	☐	☐	☐	☐

14) **Did you receive enough information about warning signs to be aware of regarding your illness/your health condition or your assistive device?**

1. Not at all	2.	3.	4. Yes, completely	5. Not applicable
☐	☐	☐	☐	☐

15) **Did the certified prosthetist & orthotist explain the treatment in a way that you understood?**

1. Not at all	2.	3.	4. Yes, completely	5. Not applicable
☐	☐	☐	☐	☐

16) **Did you get the opportunity to ask the questions you wanted?**

1. Not at all	2.	3.	4. Yes, completely	5. Not applicable
☐	☐	☐	☐	☐

17) **If you felt discomfort regarding your illness/your health condition or your treatment/assistive devices, were you met with compassion and care?**

1. Not at all	2.	3.	4. Yes, completely	5. Not applicable
☐	☐	☐	☐	☐

18) **Did you have the opportunity to receive emotional support from the healthcare staff when needed (e.g., if you felt worry, fear, anxiety, or similar)?**

1. Not at all	2.	3.	4. Yes, completely	5. Not applicable
☐	☐	☐	☐	☐

19) **Did you get enough privacy when you and the healthcare staff talked about your condition or treatment?**

1. Not at all	2.	3.	4. Yes, completely	5. Not applicable
☐	☐	☐	☐	☐

20) **If any examinations were done, did the certified prosthetist & orthotist explain the results in a way that you understood?**

1. Not at all	2.	3.	4. Yes, completely	5. Not applicable
☐	☐	☐	☐	☐

21) **Did the healthcare staff give your family/relatives the information they wanted?**

1. Not at all	2.	3.	4. Yes, completely	5. Not applicable
☐	☐	☐	☐	☐

22) **Did you experience that the healthcare staff cooperated well?**

1. Not at all	2.	3.	4. Yes, completely	5. Not applicable
☐	☐	☐	☐	☐

23) **Do you feel that your current need for care/treatment has been met?**

1. Not at all	2.	3.	4. Yes, completely	5. Not applicable
☐	☐	☐	☐	☐

24) **Did you feel well cared for and safe when you were at the Department of Prosthetics and Orthotics?**

1. Not at all	2.	3.	4. Yes, completely	5. Not applicable
☐	☐	☐	☐	☐

25) **Have you received information about where your nearest diabetes foot clinic is located?**

1. Not at all	2.	3.	4. Yes, completely	5. Not applicable
☐	☐	☐	☐	☐

26) **Did you experience that it was clean at the clinic?**

1. Not at all	2.	3.	4. Yes, completely	5. Not applicable
☐	☐	☐	☐	☐

27) **Were you satisfied with the visit as a whole at the clinic?**

1. Not at all	2.	3.	4. Yes, completely	5. Not applicable
☐	☐	☐	☐	☐

About you

28 Please state your highest completed education. Select only one answer

☐ Compulsory school, elementary school or equivalent

☐ High school, secondary school or equivalent

☐ Post-secondary education, university or college

☐ No completed education

29 Please state your main occupation. Select only one answer

☐ Employed

☐ Unemployed

☐ Student

☐ Retired

☐ Other

30 Please indicate whether your visit was a:

☐ First visit

☐ Return visit

31 It is not possible to ask everything in a survey. Do you have other viewpoints or would you like to further develop your answers? Please write clearly.

……

……

Thank you for your participation!
