# Supplementary material for: How do patients and healthcare professionals experience foot examinations in diabetes care? – A randomised controlled study of digital foot examinations versus traditional foot examinations
Source: BMC Health Serv Res. 2024 Nov 12;24:1387. doi: 10.1186/s12913-024-11674-w (PMC11558827; doi:10.1186/s12913-024-11674-w)
Supplement: Supplementary file 3 — Supplementary Material 3. Orthotics & prosthetics users survey. [file 12913_2024_11674_MOESM3_ESM.pdf]

20694

Client ID

OPUS: Satisfaction With Device and Services

Authors note: Questions 12 - 21 was used in this study.

Please mark the response that most closely reflects your opinion.

	Strongly Agree	Agree	Neither Agree nor Disagree	Disagree	Strongly Disagree	Don't Know / Not Applicable
1. My prosthesis / orthosis fits well	O	O	O	O	O	O
2. The weight of my prosthesis / orthosis is manageable	O	O	O	O	O	O
3. My prosthesis / orthosis is comfortable throughout the day	O	O	O	O	O	O
4. It is easy to put on my prosthesis / orthosis	O	O	O	O	O	O
5. My prosthesis / orthosis looks good	O	O	O	O	O	O
6. My prosthesis / orthosis is durable	O	O	O	O	O	O
7. My clothes are free of wear and tear from my prosthesis / orthosis	O	O	O	O	O	O
8. My skin is free of abrasions and irritations	O	O	O	O	O	O
9. My prosthesis / orthosis is pain free to wear	O	O	O	O	O	O
10. I can afford the out-of-pocket expenses to purchase and maintain my prosthesis / orthosis	O	O	O	O	O	O
11. I can afford to repair or replace my prosthesis / orthosis as soon as needed	O	O	O	O	O	O
12. I received an appointment with a prosthetist / orthotist within a reasonable amount of time	O	O	O	O	O	O
13. I was shown the proper level of courtesy and respect by the staff	O	O	O	O	O	O
14. I waited a reasonable amount of time to be seen	O	O	O	O	O	O
15. Clinic staff fully informed me about equipment choices	O	O	O	O	O	O
16. The prosthetist / orthotist gave me the opportunity to express my concerns regarding my equipment	O	O	O	O	O	O
17. The prosthetist / orthotist was responsive to my concerns and questions	O	O	O	O	O	O
18. I am satisfied with the training I received in the use and maintenance of my prosthesis / orthosis	O	O	O	O	O	O
19. The prosthetist / orthotist discussed problems I might encounter with my equipment	O	O	O	O	O	O
20. The staff coordinated their services with my therapists and doctors	O	O	O	O	O	O
21. I was a partner in decision-making with clinic staff regarding my care and equipment	O	O	O	O	O	O

© Rehabilitation Institute of Chicago, 2014

TILLFREDSSTÄLLELSE MED ORTOPEDTEKNISK SERVICE

Med tanke på ditt besök, hur väl stämmer följande påståenden?

	Stämmer mycket väl 1	Stämmer 2	Stämmer inte 3	Ej aktuellt
1. Jag fick en tid hos en ortopedingenjör/tekniker inom rimlig tid.	☐	☐	☐	☐
2. Personalen bemötte mig artigt och med respekt.	☐	☐	☐	☐
3. Väntetiden vid besöket var rimlig.	☐	☐	☐	☐
4. Personalen informerade mig väl om alternativa ortopedtekniska hjälpmedel.	☐	☐	☐	☐
5. Ortopedingenjören/teknikern gav mig möjlighet att uttrycka mina åsikter om hjälpmedlet.	☐	☐	☐	☐
6. Ortopedingenjören/teknikern var lyhörd för min oro och mina frågor.	☐	☐	☐	☐
7. Jag är nöjd med träningen och instruktionerna jag fått för att kunna använda och underhålla mitt hjälpmedel.	☐	☐	☐	☐
8. Ortopedingenjören/teknikern informerade om problem som jag kan tänkas få med mitt hjälpmedel.	☐	☐	☐	☐
9. Personalen samordnade sina åtgärder med min sjukgymnast, arbetsterapeut och läkare.	☐	☐	☐	☐
10. Jag var delaktig i besluten angående min utprovning och mitt hjälpmedel.	☐	☐	☐	☐

OPUS Orthotics and Prosthetics Users' Survey Copyright 2015 © Northwestern University. All Rights Reserved
Svensk översättning: Gustav Jarl och Liselotte Norling Hermansson, Region Örebro län
