# Supplementary material for: How do patients and healthcare professionals experience foot examinations in diabetes care? – A randomised controlled study of digital foot examinations versus traditional foot examinations
Source: BMC Health Serv Res. 2024 Nov 12;24:1387. doi: 10.1186/s12913-024-11674-w (PMC11558827; doi:10.1186/s12913-024-11674-w)
Supplement: Supplementary file 4 — Supplementary Material 4. Survey to healthcare professionals I. [file 12913_2024_11674_MOESM4_ESM.pdf]

Supplementary file 4. Questionnaire to certified prosthetics and orthotics/shoe technicians participating in a study of foot examinations at the department of prosthetics and orthotics and the implementation of D-Foot

The questionnaire contains questions on foot examinations you have performed on patients with diabetes and foot complications. Answer the questions by marking the answer that is most suitable. If you are unsure, you should still mark the answer that feels most correct. Put a cross in the square like this ☒

How was the interaction with the patient?

Answer the questions by marking the answer that is most suitable on a scale of 1 (very poor or not at all) to 4 (excellent or yes, completely).
If you are unsure, you should still mark the answer that feels most correct. Put a cross in the square like this ☒

11) **Was the patient given advice on self-care for his/her feet?**

1. Not at all	2.	3.	4. Yes, completely	5. Not relevant
☐	☐	☐	☐	☐

12) **Was the patient's illness/health status discussed with the patient?**

1. Not at all	2.	3.	4. Yes, completely	5. Not relevant
☐	☐	☐	☐	☐

13) **Did you give the patient information about who to consult if he/she needed help or if other questions arose after the visit?**

1. Not at all	2.	3.	4. Yes, completely	5. Not relevant
☐	☐	☐	☐	☐

14) **Did you give the patient information about possible risks associated with using the device/equipment?**

1. Not at all	2.	3.	4. Yes, completely	5. Not relevant

☐ ☐ ☐ ☐ ☐

15) **Did you give the patient information about warning signals to watch out for in association with his/her illness/health status or the device/equipment?**

1. Not at all	2.	3.	4. Yes, completely	5. Not relevant
☐	☐	☐	☐	☐

16) **Were you and the patient sufficiently isolated when the patient's condition/status or treatment were discussed?**

1. Not at all	2.	3.	4. Yes, completely	5. Not relevant
☐	☐	☐	☐	☐

17) **Did you explain the result of the D-Foot examination to the patient?**

1. Not at all	2.	3.	4. Yes, completely	5. Not relevant
☐	☐	☐	☐	☐

18) **Did the patient receive oral user information?**

1. Not at all	2.	3.	4. Yes, completely	5. Not relevant
☐	☐	☐	☐	☐

19) **Did the patient receive written user information?**

1. Not at all	2.	3.	4. Yes, completely	5. Not relevant
☐	☐	☐	☐	☐

25) **Do you have any suggestions for ways of improving the D-Foot web program?**

- ☐ Yes
- ☐ No

24) **Have you previously used any web program as support during foot examinations?**

- ☐ Yes
- ☐ No

20) **How long did the visit last? Do not include the time taken to record the visit in Pilot.**

- ☐ Less than 30 minutes
- ☐ 31-45 minutes
- ☐ 46-60 minutes
- ☐ More than 60 minutes

21) **How long did it take to record the visit in Pilot?**

- ☐ Less than 5 minutes
- ☐ 6-10 minutes
- ☐ 11-15 minutes
- ☐ 16-20 minutes

22) **How long did it take to make any shoe and material orders?**

- ☐ Less than 5 minutes
- ☐ 6-10 minutes
- ☐ 11-15 minutes
- ☐ 16-20 minutes

Part III: About you

23) **How much experience do you have as an certified prosthetics and orthotics/shoe technicians ?**

- ☐ Less than 1 year
- ☐ 1-5 years
- ☐ 5-9 years
- ☐ 10-20 years
- ☐ More than 20 years

26. If you have answered Yes to question 25, you can explain your answer in more detail here. Please write clearly.

……
……
……
……
……
……
……
……
……
……
……
……

27. It is impossible to ask everything in a questionnaire, Do you have any other views or would you like to extend your answers? Please write clearly.

……
……
……
……
……
……
……
……
……
……
……
……
