# Supplementary material for: How do patients and healthcare professionals experience foot examinations in diabetes care? – A randomised controlled study of digital foot examinations versus traditional foot examinations
Source: BMC Health Serv Res. 2024 Nov 12;24:1387. doi: 10.1186/s12913-024-11674-w (PMC11558827; doi:10.1186/s12913-024-11674-w)
Supplement: Supplementary file 5 — Supplementary Material 5. Survey to healthcare professionals. [file 12913_2024_11674_MOESM5_ESM.pdf]

Supplementary file 5. Addition to the questionnaire to certified prosthetics and orthotics/shoe technicians participating in a study of foot examinations at Orthopaedic Technology and the implementation of D-Foot

The questionnaire contains questions on foot examinations you have performed on patients with diabetes and foot complications. Answer the questions by marking the answer that is most suitable. If you are unsure, you should still mark the answer that feels most correct. Put a cross in the square like this ☒

21a) **How long did it take to record the visit in the medical record system (Pilot) when you examined the feet using the D-Foot method?**

☐ Less than 5 minutes

☐ 6-10 minutes

☐ 11-15 minutes

☐ 16-20 minutes

21b) **How long did it take to record the visit in Pilot when you examined the feet in the established way?**

☐ Less than 5 minutes

☐ 6-10 minutes

☐ 11-15 minutes

☐ 16-20 minutea
